# Annual and Analytical Cyclopedia of Practical Medicine

**Published:** 1900-11

**Authors:** 


ANNUAL AND ANALYTICAL CYCLOPEDIA OF PRACTICAL MEDICINE. By Charles E. de M. Sajous, M. D., and one hundred associate editors, assisted by corresponding editors, collaborators and correspondents. Illustrated with chromo-lithograph engravings and maps. Volume V. Pages, 662; price, $3.00. Philadelphia, New York, Chicago: The F. A. Davis Co., Publishers. 1900.

This annual embraces the current medical literature of the world. It is arranged in cyclopedic or dictionary form, without index, and in many ways is an improvement over the five volume work of a few years since by the same editor.

Physicians who have kept up with this work and have all the volumes have a fine library, and each year thereafter adds the current progress to the collection.

